# Prolonged direct hemoperfusion using a polymyxin B immobilized fiber cartridge provides sustained circulatory stabilization in patients with septic shock: a retrospective observational before-after study

**DOI:** 10.1186/s40560-017-0214-3

**Published:** 2017-02-20

**Authors:** Kyohei Miyamoto, Yu Kawazoe, Seiya Kato

**Affiliations:** 1 Department of Emergency and Critical Care Medicine, Wakayama Medical University, 811-1, Kimiidera, Wakayama City, Wakayama Japan; 2 Division of Emergency and Critical Care Medicine, Tohoku University Hospital Emergency Center, 1-1, Seiryo-machi, Aoba-ku, Sendai City, Miyagi Japan

**Keywords:** PMX-DHP, Treatment duration, Septic shock, Hemodynamics

## Abstract

**Background:**

Direct hemoperfusion therapy with polymyxin B immobilized fiber cartridges (PMX-DHP) is widely used for septic shock in Japan and parts of Europe. Although this treatment is usually administered for 2 h, the optimal duration has not been established.

**Methods:**

This retrospective study compared the effects of prolonged and conventional PMX-DHP durations (2 and 12 h, respectively) for septic shock. Between October 2013 and March 2015, 18 patients underwent conventional PMX-DHP, and between April 2015 and May 2016, 18 patients underwent prolonged PMX-DHP. The primary outcome was the vasopressor dependency index during the 12 h after starting the first PMX-DHP session. The vasopressor dependency index was calculated as (inotropic score)/(mean blood pressure).

**Results:**

The patients’ characteristics were almost similar in the conventional and prolonged PMX-DHP groups. The major site of infection was the abdomen in both groups (61 and 72%, respectively). The conventional PMX-DHP group had mean blood pressure values of 68.4 ± 8.9 mmHg and 78.2 ± 16.9 mmHg at 0 and 12 h after starting PMX-DHP (*P* = 0.13). The prolonged PMX-DHP group had mean blood pressure values of 70.3 ± 15.7 mmHg and 87.7 ± 16.9 mmHg at 0 and 12 h after starting PMX-DHP (*P* = 0.004). The conventional PMX-DHP group had vasopressor dependency index values of 0.52 ± 0.29 and 0.39 ± 0.25 at 0 and 12 h after starting PMX-DHP (*P* = 0.29). The prolonged PMX-DHP group had vasopressor dependency index values of 0.50 ± 0.26 and 0.28 ± 0.18 at 0 and 12 h after starting PMX-DHP (*P* = 0.01). Hospital mortality was similar in both groups (8/18 [44%] and 8/18 [44%]).

**Conclusions:**

These findings suggest that prolonged PMX-DHP provides more sustained circulatory stabilization compared to conventional PMX-DHP. However, our study failed to detect any improvement in mortality. Well-designed prospective trials are needed to examine the clinical outcomes of prolonged PMX-DHP and to identify the optimal duration of PMX-DHP.

**Electronic supplementary material:**

The online version of this article (doi:10.1186/s40560-017-0214-3) contains supplementary material, which is available to authorized users.

## Background

Septic shock is the most severe form of infection and the related mortality rate is >40% [1]. Endotoxins play a key role in the deterioration of patients with sepsis and can induce organ failure or shock. Endotoxemia may be a specific therapeutic target for treating sepsis, and patients with septic shock may undergo endotoxin hemadsorption using direct hemoperfusion therapy with polymyxin B immobilized fiber cartridges (PMX-DHP). Since the early 2000s, several randomized controlled trials of PMX-DHP revealed that it increased the patients’ blood pressure [2, 3] but had inconsistent effects on the mortality rate [2–4].

The conventional duration of PMX-DHP is 2 h, although a recent retrospective observational study found that endotoxin removal also occurred at >2 h during prolonged PMX-DHP [5]. Furthermore, a prospective observational study by Mitaka et al. revealed that prolonged PMX-DHP for septic shock provided a greater reduction of the required noradrenaline dose and sequential organ failure assessment (SOFA) score compared to conventional PMX-DHP [6]. However, no other clinical studies have compared prolonged and conventional PMX-DHP, and the clinical efficacy of prolonged PMX-DHP is unknown. Therefore, the present study aimed to compare prolonged and conventional PMX-DHP among patients with septic shock, based on the resulting blood pressure elevation, vasopressor dependency index, and mortality rate.

## Methods

This retrospective observational before-after study compared the effects of conventional and prolonged PMX-DHP among patients with septic shock. The study’s design was approved by the institutional review board of Wakayama Medical University, which waived the requirement for informed consent because of the retrospective and observational nature of the study.

The patients for this study were treated in a medical-surgical intensive care unit (ICU) at Wakayama Medical University, which has 10 beds and a closed-ICU system. At our institution, PMX-DHP with Toraymyxin (PMX-20R; Toray Industries, Tokyo, Japan) is used for patients with septic shock who require high-dose vasopressor treatment (>0.1 μg/kg/min of noradrenaline). Between October 2013 and March 2015, 19 patients underwent 2 h of conventional PMX-DHP (the PMX-DHP-2 h group), and between April 2015 and May 2016, 18 patients underwent 12 h of prolonged PMX-DHP (the PMX-DHP-12 h group). However, we excluded one patient from the PMX-DHP-2 h group who died within 24 h after the first PMX-DHP session, and only 36 patients were included in the analyses. Other management modalities for sepsis were unchanged throughout the study period and were based on the Surviving Sepsis Campaign Guidelines [7] and the Japanese guidelines for sepsis management [8].

The patients’ medical records were retrospectively evaluated to retrieve data for the analyses. The primary outcome was the vasopressor dependency index within 12 h after starting the first PMX-DHP session. The secondary outcomes were the mean blood pressure, the inotropic score within 12 h after starting the first PMX-DHP session, ICU-free days at day 28, vasopressor-free days at day 28, renal replacement therapy (RRT)-free days at day 28, ventilator-free days at day 28, ICU mortality, hospital mortality, and the SOFA score during the week after the first PMX-DHP session. The inotropic score was calculated as previously described [2]: (dopamine dose [μg/kg/min]) + (dobutamine dose [μg/kg/min]) + (adrenaline dose [μg/kg/min]) × 100 + (noradrenaline dose [μg/kg/min]) × 100 + (phenylephrine dose [μg/kg/min]) × 100. The vasopressor dependency index was also calculated as previously described [2]: (inotropic score)/(mean blood pressure). The numbers of ICU-free, vasopressor-free, RRT-free, and ventilator-free days at day 28 were calculated by subtracting the number of days that the patients spent in the ICU, under vasopressor use, under RRT, or under ventilation after the first PMX-DHP session. Patients who died before day 28 were assigned values of 0 for their ICU-free, vasopressor-free, RRT-free, and ventilator-free days. The calculations for vasopressor-free days considered dopamine, adrenaline, and noradrenaline as vasopressors. The SOFA scores were calculated for each patient who was alive at the post-treatment time points. The data for arterial blood gas analysis including serum lactate levels and the PaO_2_/FiO_2_ ratio were evaluated at 0, 2, 5, 8, and 12 h. For the collection of arterial blood gas analysis data, the gap of actual sampling time ±1 h was tolerated.

### Statistical analysis

Continuous variables were presented as mean ± standard deviation or median and interquartile range (IQR). Categorical variables were presented as number and percentage (%). The PMX-DHP-2 h and PMX-DHP-12 h groups were compared using the Wilcoxon rank sum test for continuous variables and Fisher’s exact test for categorical variables. Mean blood pressures, vasopressor dependency indexes, inotropic scores, the serum lactate, and PaO_2_/FiO_2_ ratio were analyzed by analysis of variance following Dunnett’s test, with the reference value being the baseline value (0 h, before starting the first PMX-DHP session). Vasopressor dependency indexes were compared between groups using two-way repeated measurements analysis of variance. A two-sided *P* value of <0.05 was considered statistically significant, and all analyses were performed using JMP Pro software (version 12.2; SAS Institute Inc., Cary, NC, USA).

## Results

The patients’ characteristics are presented in Table 1. The two groups were generally similar (no statistically significant differences), with the exception of median serum lactate levels being significantly higher in the PMX-DHP-2 h group (PMX-DHP-2 h: 7.0 mmol/L [IQR: 4.7–11.7 mmol/L]; PMX-DHP-12 h: 4.6 mmol/L (IQR: 2.0–7.6 mmol/L); *P* = 0.03). The PMX-DHP-2 h group had 18 patients (100%) with serum lactate levels of >2 mmol/L, compared to 13 patients (72%) in the PMX-DHP-12 h group (*P* = 0.046). Nine patients (50%) in the PMX-DHP-2 h group and 11 patients (61%) in the PMX-DHP-12 h group were admitted to the ICU from the operating room after emergency surgery. Among these patients, the median times from emergency department admission to operating room entrance were 1 h (IQR 1–2 h) in the PMX-DHP-2 h group and 2 h (IQR 1–6 h) in the PMX-DHP-12 h group (*P* = 0.1).

**Table 1:** Patients’ characteristics

	PMX-DHP-2 h (*n* = 18)	PMX-DHP-12 h (*n* = 18)	*P* value
Age, years, median [IQR]	75 (64–84)	78 (60–83)	0.75
Male, number (%)	10 (56%)	7 (39%)	0.51
Body weight, kg, median [IQR]	60 (54–65)	58 (50–64)	0.45
APACHE II score at ICU admission, median [IQR]	25 (19–31)	21 (18–30)	0.40
Comorbidity			
Immunosuppression, number (%)	4 (22%)	4 (22%)	1.00
Liver cirrhosis, number (%)	2 (11%)	0 (0%)	0.49
Chronic dialysis, number (%)	0 (0%)	2 (11%)	0.49
Home oxygenation therapy, number (%)	0 (0%)	1 (6%)	1.00
Decompensate heart failure, number (%)	0 (0%)	1 (6%)	1.00
Source of admission to ICU			0.81
Operating room after emergency surgery, number (%)	9 (50%)	11 (61%)	
Emergency department, number (%)	6 (33%)	5 (28%)	
Hospital ward, number (%)	3 (17%)	2 (11%)	
Site of infection			
Abdomen, number (%)	11 (61%)	13 (72%)	0.72
Thorax, number (%)	2 (11%)	1 (6%)	0.61
Skin and soft tissue, number (%)	2 (11%)	1 (6%)	0.61
Urinary tract, number (%)	1 (6%)	2 (11%)	0.61
Others, number (%)	1 (6%)	2 (11%)	0.61
Unknown, number (%)	1 (6%)	0 (0%)	1.00
Causative microorganisms			
Gram-negative rods, number (%)	4 (22%)	2 (11%)	0.66
Gram-positive cocci, number (%)	4 (22%)	3 (17%)	1.00
Gram-positive rods, number (%)	1 (6%)	0 (0%)	1.00
Fungus, number (%)	0 (0%)	1 (6%)	1.00
Mixed, number (%)	3 (17%)	7 (39%)	0.26
Unknown, number (%)	6 (33%)	5 (28%)	1.00
Number of PMX-DHP session			1.00
1 session, number (%)	6 (33%)	7 (39%)	
2 sessions, number (%)	12 (67%)	11 (61%)	
Start of PMX-DHP from the onset of shock or ICU admission, h, median [IQR]^a^	3 (2–4)	4 (3–5)	0.09
Treatment in ICU			
Continuous renal replacement therapy, number (%)	11 (61%)	8 (44%)	0.51
Mechanical ventilation, number (%)	18 (100%)	18 (100%)	1.00
Low-dose steroid, number (%)	17 (94%)	15 (83%)	0.60
Dose of fluid administration within 24 h after starting PMX-DHP, ml, median [IQR]	4285 [3587–7207]	4780 [3463–8220]	0.69
Laboratory test^b^			
White blood cell, 10^2^/mcl, median [IQR]	77.0 (34.0–172.5)	88.2 (47.0–146.3)	1.00
C reactive protein, mg/dl, median [IQR]	13.5 (4.6–22.5)	15.9 (9.2–24.1)	0.28
Total bilirubin, mg/dl, median [IQR]	1.1 (0.8–2.1)	0.8 (0.6–1.8)	0.20
Creatinine, mg/dl, median [IQR]	1.8 (1.1–2.5)	1.6 (1.2–2.5)	0.78
PT-INR, median [IQR]	1.3 (1.2–2.0)	1.3 (1.2–1.8)	0.94
Lactate, mmol/l, median [IQR]	7.0 (4.7–11.7)	4.6 (2.0–7.6)	0.03

*PMX-DHP-2 h* polymyxin direct hemoperfusion for 2 h, *PMX-DHP-12 h* polymyxin direct hemoperfusion for 12 h, *IQR* interquartile range, *APACHE II* acute physiology and chronic health evaluation, *ICU* intensive care unit, *PT-INR* prothrombin time-international normalized ratio

^a^Onset of shock was defined as the timing of inotropic score >10

^b^Laboratory test was performed at the day of first PMX-DHP session

**Fig. 1:**
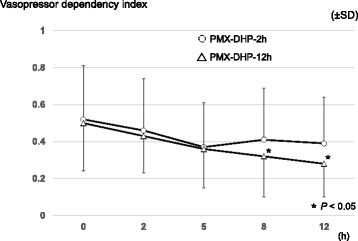
Vasopressor dependency index after the first PMX-DHP session. Multiple comparisons were performed in each group, using Dunnett’s test, with the baseline values as the reference values (0 h; before starting the first PMX-DHP session). PMX-DHP-2 h: polymyxin direct hemoperfusion for 2 h, PMX-DHP-12 h: polymyxin direct hemoperfusion for 12 h, SD: standard deviation

The primary outcome is shown in Fig. 1 and Table 2. In the PMX-DHP-12 h group, the vasopressor dependency index values were significantly decreased at 8 h (*P* = 0.038) and 12 h (*P* = 0.011). There were no significant differences in the time courses for vasopressor dependency index when we compared the PMX-DHP-2 h group and PMX-DHP-12 h group (*P* = 0.52).

**Table 2:** The time course of the vasopressor dependency index, the mean blood pressure, and inotropic score within 12 h after starting the first PMX-DHP session

	0 h (baseline)	2 h	5 h	8 h	12 h
Vasopressor dependency index, mean ± SD					
PMX-DHP-2 h (*n* = 18)	0.52 ± 0.29	0.46 ± 0.28	0.37 ± 0.24	0.41 ± 0.28	0.39 ± 0.25
PMX-DHP-12 h (*n* = 18)	0.50 ± 0.26	0.43 ± 0.20	0.36 ± 0.21	0.32 ± 0.22^*^	0.28 ± 018^*^
Mean blood pressure, mmHg, mean ± SD					
PMX-DHP-2 h (*n* = 18)	68.4 ± 8.9	76.9 ± 14.8	83.4 ± 14.2^*^	75.6 ± 14.9	78.2 ± 16.9
PMX-DHP-12 h (*n* = 18)	70.3 ± 15.7	80.7 ± 13.8	86.4 ± 15.6^*^	87.0 ± 18.4^*^	87.7 ± 16.9^*^
Inotropic score, mean ± SD					
PMX-DHP-2 h (*n* = 18)	33.9 ± 17.3	33.3 ± 18.0	28.4 ± 14.8	27.4 ± 15.1	27.3 ± 15.4
PMX-DHP-12 h (*n* = 18)	33.8 ± 14.8	34.6 ± 15.9	29.9 ± 14.1	24.7 ± 13.8	23.1 ± 13.1

*P < 0.05 (Dunnett’s test with the reference value being the 0 h value)

The mean blood pressure and the inotropic scores were also shown in Table 2. In the PMX-DHP-2 h group, the mean blood pressure value was significantly elevated at 5 h (*P* = 0.01), although it was not statistically significant at 8 h (*P* = 0.37) and 12 h (*P* = 0.13). In the PMX-DHP-12 h group, the mean blood pressure values were significantly elevated at 5 h (*P* = 0.009), 8 h (*P* = 0.006), and 12 h (*P* = 0.004). The detailed values for each inotrope are shown in Additional file 1: Table S1.

The values for urinary output did not differ significantly within 24 h after starting PMX-DHP (Additional file 1: Table S2). The time courses of the serum lactate and PaO_2_/FiO_2_ ratio within 12 h after starting PMX-DHP are shown in Additional file 1: Table S3. In each group, the statistically significant changes were not observed. The SOFA scores during the week after the first PMX-DHP session are shown in Additional file 1: Table S4, although no significant inter-group differences were observed at days 1, 2, 3, and 7. The other secondary outcomes are shown in Table 3. The PMX-DHP-12 h group had higher numbers of ICU-free, vasopressor-free, RRT-free, and ventilator-free days, compared to the PMX-DHP-2 h group, although these differences were not statistically significant (ICU-free: 18 vs. 7 days, *P* = 0.31; vasopressor-free: 5 vs. 21 days, *P* = 0.11; RRT-free: 2 vs. 20 days, *P* = 0.31; ventilator-free: 8 vs. 3 days, *P* = 0.22). Both groups also had similar ICU mortality and hospital mortality rates. None of the patients developed complications that were related to the PMX-DHP, including coagulation in the circuit.

**Table 3:** Secondary outcomes

	PMX-DHP-2 h (*n* = 18)	PMX-DHP-12 h (*n* = 18)	*P* value
ICU-free days at day 28, days, median [IQR]	7 (0–23)	18 (0–24)	0.31
Vasopressor-free days at day 28, days, median [IQR]	5 [0–23]	21 [0–24]	0.11
RRT-free days at day 28, days, median [IQR]	2 [0–28]	20 [0–28]	0.31
Ventilator-free days at day 28, days, median [IQR]	3 (0–22)	18 (0–25)	0.22
ICU mortality, number (%)	7 (39%)	3 (17%)	0.26
Hospital mortality, number (%)	8 (44%)	8 (44%)	1.00

*PMX-DHP-2 h* polymyxin direct hemoperfusion for 2 h, *PMX-DHP-12 h* polymyxin direct hemoperfusion for 12 h, *ICU* intensive care unit, *RRT* renal replacement therapy, *IQR* interquartile range

## Discussion

The present study revealed that prolonged PMX-DHP provided a persistent increase in blood pressure among patients with septic shock. In contrast, patients who received conventional PMX-DHP only experienced a significant increase in mean blood pressure at 5 h after starting treatment, and this significant increase was subsequently lost. Similar effects were observed for the vasopressor dependency index. Interestingly, an in vitro study using calf serum revealed that endotoxin adsorption capacity decreased over time and reached a nadir at approximately 2 h [9]. Thus, PMX-DHP is generally performed for 2 h, according to the manufacturer’s instruction. However, a recent retrospective observational study of 19 patients revealed that clinical endotoxin removal was continuously observed during 24 h of prolonged PMX-DHP [5]. Unfortunately, there are very few other studies regarding the clinical effectiveness of prolonged PMX-DHP. One case series of five patients with *Staphylococcus aureus* toxic shock syndrome found that prolonged PMX-DHP significantly increased systolic blood pressure (from 89 to 125 mmHg) and decreased the vasopressor requirement (duration 9 h, range 4–20 h) [10]. A retrospective observational study by Yamashita et al. also revealed that prolonged PMX-DHP (15.8 ± 7.9 h) significantly increased the mean blood pressure and PaO_2_/FiO_2_ ratio values among patients with severe sepsis and septic shock [11]. However, these studies are limited by their single-arm designs and cannot conclusively demonstrate that prolonged PMX-DHP is superior to conventional PMX-DHP.

Mitaka et al. performed a prospective observational study of 16 patients with septic shock and reported that prolonged PMX-DHP (11 patients, 16.9 ± 7.0 h) provided a greater reduction of the required noradrenaline dose compared to conventional PMX-DHP (five patients, 2 h) [6]. The mean change in the noradrenaline dose (before and after the PMX-DHP session) was –17.8 ± 14.6 μg/min in the prolonged PMX-DHP group compared to –1.8 ± 2.7 μg/min in the conventional PMX-DHP group (*P* < 0.05). However, that study compared different time points in the two groups, rather fixed time points, and the interval to the outcome evaluation was longer in the prolonged PMX-DHP group. Thus, these data are insufficient to determine whether prolonged or conventional PMX-DHP have greater effectiveness.

The strength of the present study is its comparison of prolonged and conventional PMX-DHP at fixed time points, which allowed us to detect a sustained increase in blood pressure and decrease in vasopressor dependency index among the PMX-DHP-12 h group, but not in the PMX-DHP-2 h group. Thus, prolonged PMX-DHP may provide greater improvements in the patient’s short-term circulatory status. Furthermore, the present study demonstrated nonsignificant improvements in some secondary outcomes, such as vasopressor-free days and ventilator-free days. Sustained circulatory stabilization might improve these clinical outcomes.

The present study also has several limitations. First, we did not perform a randomized controlled trial, and we cannot confirm that the groups’ patient characteristics were adequately balanced. For example, serum lactate levels were significantly higher in the PMX-DHP-2 h group than in the PMX-DHP-12 h group, although the similar Acute Physiology and Chronic Health Evaluation II (APACHE II) scores suggested that any inter-group differences in severity were relatively small. Nevertheless, serum lactate levels are an important predictive factor in cases of septic shock [1], and the PMX-DHP-2 h group may have included more severe cases. Second, our study only evaluated the circulatory index for a short 12-h period after starting the first PMX-DHP session, which may explain the discrepancy between our findings and those from previous studies that observed increased blood pressure values at 2–3 days after conventional PMX-DHP [2, 3]. Thus, it may be desirable to evaluate mean blood pressure over a longer period, although this would be difficult to interpret in our study, because mean blood pressure would be markedly biased by the timing of the next PMX-DHP session (which may start at 12 h after the first session). Thus, we compared the SOFA scores over the first 7 days and there were no significant differences in organ dysfunction and circulatory failure. Third, approximately 20% of our patients had Gram-positive infections, which might decrease the effectiveness of PMX-DHP and result in our analyses being underpowered. However, a previous study found elevated endotoxin levels in cases of both Gram-negative and Gram-positive infections [12]. In addition, PMX-DHP has been reported to have a secondary effect of removing activated monocytes and neutrophils [13]. Thus, PMX-DHP may still provide beneficial effects in patients with Gram-positive infections. Furthermore, the five-patient case series found that blood pressure was increased using prolonged PMX-DHP for patients with Gram-positive infections [10]. Fourth, our study only included a small number of patients and was underpowered to detect differences in clinical outcomes, such as mortality. Therefore, larger comparative studies are needed to assess the true value of prolonged PMX-DHP.

## Conclusions

This retrospective before-after study compared conventional and prolonged PMX-DHP among patients with septic shock and revealed that prolonged PMX-DHP provided sustained improvements in blood pressure and vasopressor dependency index values during the first 12 h. However, our study failed to detect any improvement in mortality, which was likely related to the small sample size. Well-designed prospective trials are needed to examine the clinical outcomes of prolonged PMX-DHP and to identify the optimal duration of PMX-DHP.

## Additional file

Additional file 1: Table S1. The catecholamine usage and dosing within 12 h from starting PMX-DHP. **Table S2.** Time course of the urine output within 12 h after starting PMX-DHP. **Table S3.** Time course of the serum lactate and PaO_2_/FiO_2_ ratio within 12 h after starting PMX-DHP. **Table S4.** The SOFA score during one week after the first PMX-DHP session. (DOCX 27 kb)

## Abbreviations

APACHE II: Acute Physiology and Chronic Health Evaluation II
ICU: Intensive care unit
IQR: Interquartile range
PMX-DHP: Direct hemoperfusion therapy with polymyxin B immobilized fiber cartridge
PT-INR: Prothrombin time-international normalized ratio
RRT: Renal replacement therapy
SD: Standard deviation
SOFA: Sequential organ failure assessment
