# Supplementary material for: Prolonged direct hemoperfusion using a polymyxin B immobilized fiber cartridge provides sustained circulatory stabilization in patients with septic shock: a retrospective observational before-after study
Source: J Intensive Care. 2017 Feb 20;5:19. doi: 10.1186/s40560-017-0214-3 (PMC5319036; doi:10.1186/s40560-017-0214-3)
Supplement: Additional file 1: Table S1. — The catecholamine usage and dosing within 12 h from starting PMX-DHP. Table S2. Time course of the urine output within 12 h after starting PMX-DHP. Table S3. Time course of the serum lactate and PaO2/FiO2 ratio within 12 h after starting PMX-DHP. Table S4. The SOFA score during one week after the first PMX-DHP session. (DOCX 27 kb) [file 40560_2017_214_MOESM1_ESM.docx]

Additional file 1

**Table S1** The catecholamine usage and dosing within 12 h from starting PMX-DHP

|  | 0 h (Baseline) | 2 h | 5 h | 8 h | 12 h |
| --- | --- | --- | --- | --- | --- |
| Dopamine |  |  |  |  |  |
| Usage, N (%) |  |  |  |  |  |
| PMX-DHP-2h (n = 18) | 17 (94%) | 17 (94%) | 17 (94%) | 17 (94%) | 17 (94%) |
| PMX-DHP-12h (n = 18) | 10 (56%) | 10 (56%) | 10 (56%) | 10 (56%) | 8 (44%) |
| Dose, µg/kg/min, median [IQR] |  |  |  |  |  |
| PMX-DHP-2h (n = 18) | 7.74 [5.20 – 9.33] | 7.48 [5.00 – 9.33] | 6.57 [4.79 – 9.33] | 6.57 [3.78 – 9.33] | 6.51 [3.66 – 9.71] |
| PMX-DHP-12h (n = 18) | 2.79 [0 – 6.61] | 2.79 [0 – 7.10] | 1.36 [0 – 7.04] | 1.36 [0 – 7.04] | 0 [0 – 5.38] |
| Dobutamine |  |  |  |  |  |
| Usage, N (%) |  |  |  |  |  |
| PMX-DHP-2h (n = 18) | 2 (11%) | 2 (11%) | 3 (17%) | 4 (22%) | 6 (33%) |
| PMX-DHP-12h (n = 18) | 5 (28%) | 5 (28%) | 5 (28%) | 5 (28%) | 5 (28%) |
| Dose, µg/kg/min, median [IQR] |  |  |  |  |  |
| PMX-DHP-2h (n = 18) | 0 [0 – 0] | 0 [0 – 0] | 0 [0 – 0] | 0 [0 – 0.59] | 0 [0 – 3.01] |
| PMX-DHP-12h (n = 18) | 0 [0 – 2.39] | 0 [0 – 2.39] | 0 [0 – 2.39] | 0 [0 – 2.39] | 0 [0 – 2.39] |
| Adrenaline |  |  |  |  |  |
| Usage, N (%) |  |  |  |  |  |
| PMX-DHP-2h (n = 18) | 4 (22%) | 4 (22%) | 4 (22%) | 4 (22%) | 4 (22%) |
| PMX-DHP-12h (n = 18) | 1 (6%) | 1 (6%) | 1 (6%) | 1 (6%) | 1 (6%) |
| Dose, µg/kg/min, median [IQR] |  |  |  |  |  |
| PMX-DHP-2h (n = 18) | 0 [0 – 0] | 0 [0 – 0.01] | 0 [0 – 0.01] | 0 [0 – 0.01] | 0 [0 – 0.01] |
| PMX-DHP-12h (n = 18) | 0 [0 – 0] | 0 [0 – 0] | 0 [0 – 0] | 0 [0 – 0] | 0 [0 – 0] |
| Noradrenaline |  |  |  |  |  |
| Usage, N (%) |  |  |  |  |  |
| PMX-DHP-2h (n = 18) | 17 (94%) | 17 (94%) | 16 (89%) | 16 (89%) | 16 (89%) |
| PMX-DHP-12h (n = 18) | 18 (100%) | 18 (100%) | 18 (100%) | 17 (94%) | 15 (83%) |
| Dose, µg/kg/min, median [IQR] |  |  |  |  |  |
| PMX-DHP-2h (n = 18) | 0.20 [0.10 – 0.30] | 0.20 [0.10 – 0.30] | 0.20 [0.10 – 0.30] | 0.18 [0.10 – 0.30] | 0.18 [0.09 – 0.30] |
| PMX-DHP-12h (n = 18) | 0.28 [0.19 – 0.37] | 0.29 [0.19 – 0.37] | 0.25 [0.15 – 0.31] | 0.20 [0.10 – 0.25] | 0.18 [0.09 – 0.25] |
| Vasopressin |  |  |  |  |  |
| Usage, N (%) |  |  |  |  |  |
| PMX-DHP-2h (n = 18) | 1 (6%) | 1 (6%) | 1 (6%) | 1 (6%) | 1 (6%) |
| PMX-DHP-12h (n = 18) | 1 (6%) | 2 (11%) | 2 (11%) | 2 (11%) | 2 (11%) |
| Dose, µg/kg/min, median [IQR] |  |  |  |  |  |
| PMX-DHP-2h (n = 18) | 0 [0 – 0] | 0 [0 – 0] | 0 [0 – 0] | 0 [0 – 0] | 0 [0 – 0] |
| PMX-DHP-12h (n = 18) | 0 [0 – 0] | 0 [0 – 0] | 0 [0 – 0] | 0 [0 – 0] | 0 [0 – 0] |

Phenylephrine was administered in no cases.

PMX-DHP-2h: polymyxin direct hemoperfusion for two hours, PMX-DHP-12h: polymyxin direct hemoperfusion for twelve hours, IQR: interquartile range

**Table S2** Time course of the urine output within 12 h after starting PMX-DHP

|  | PMX-DHP-2h | PMX-DHP-12h | *P* value |
| --- | --- | --- | --- |
| 0 h, ml/h, median [IQR] | 31 [12 – 81] | 51 [21 – 80] | 0.58 |
| 2 h, ml/h, median [IQR] | 59 [19 – 169] | 68 [25 – 106] | 0.61 |
| 5 h, ml/h, median [IQR] | 43 [11 – 131] | 83 [15 – 125] | 0.90 |
| 8 h, ml/h, median [IQR] | 49 [5 – 105] | 87 [11 – 126] | 0.45 |
| 12 h, ml/h, median [IQR] | 37 [21 – 61] | 74 [29 – 102] | 0.13 |

PMX-DHP-2h: polymyxin direct hemoperfusion for two hours, PMX-DHP-12h: polymyxin direct hemoperfusion for twelve hours, IQR: interquartile range

**Table S3** Time course of the serum lactate and PaO_2_/FiO_2_ ratio within 12 h after starting PMX-DHP

|  | 0 h (Baseline)^#^ | 2 h | 5 h | 8 h | 12 h |
| --- | --- | --- | --- | --- | --- |
| Number of measured patients, N (%)^$^ |  |  |  |  |  |
| PMX-DHP-2h (n = 18) | 18 (100%) | 12 (67%) | 10 (56%) | 10 (56%) | 11 (%) |
| PMX-DHP-12h (n = 18) | 18 (100%) | 17 (94%) | 13 (%) | 15 (%) | 14 (%) |
| Serum lactate, mmol/l, median [IQR] |  |  |  |  |  |
| PMX-DHP-2h (n = 18) | 4.8 [2.4 – 8.1] | 5.1 [3.1 – 9.6] | 5.8 [4.4 – 12.0] | 7.0 [3.3 – 10.9] | 5.6 [3.5 – 8.6] |
| PMX-DHP-12h (n = 18) | 2.9 [1.3 – 5.7] | 3.1 [1.6 – 5.9] | 2.3 [1.7 – 3.7] | 2.3 [1.8 – 3.3] | 1.9 [1.6 – 3.1] |
| PaO_2_/FiO_2_ ratio, median [IQR] |  |  |  |  |  |
| PMX-DHP-2h (n = 18) | 197 [119 – 297] | 235 [179 – 270] | 245 [157 – 306] | 264 [175 – 307] | 222 [160 – 313] |
| PMX-DHP-12h (n = 18) | 188 [138 – 298] | 223 [150 – 312] | 222 [122 – 339] | 268 [160 – 383] | 252 [166 – 350] |

# Timing of arterial blood gas analysis was calculated from starting the first PMX-DHP session.

$ For the collection of arterial blood gas analysis data, the gap of actual sampling time ± one hour was tolerated.

PMX-DHP-2h: polymyxin direct hemoperfusion for two hours, PMX-DHP-12h: polymyxin direct hemoperfusion for twelve hours, IQR: interquartile range

**Table S4** The SOFA score during one week after the first PMX-DHP session

|  | PMX-DHP-2h  (n=18) | PMX-DHP-12h  (n=18) | *P* value |
| --- | --- | --- | --- |
| Day 1, number (%) | 18 (100%) | 18 (100%) |  |
| Respiratory score, median [IQR] | 3 (2-3) | 3 (2-3) | 0.66 |
| Coagulation score, median [IQR] | 2 (0-2) | 1 (1-2) | 0.99 |
| Hepatic score, median [IQR] | 1 (0-2) | 0 (0-1) | 0.40 |
| Cardiovascular score, median [IQR] | 4 (3-4) | 4 (3-4) | 0.60 |
| Neurological score, median [IQR] | 0 (0-2) | 1 (0-2) | 0.53 |
| Renal score, median [IQR] | 1 (1-3) | 2 (0-3) | 0.88 |
| Total score, median [IQR] | 11 (9-14) | 9 (8-13) | 0.42 |
| Day 2, number (%) | 18 (100%) | 18 (100%) |  |
| Respiratory score, median [IQR] | 2 (2-3) | 2 (1-3) | 0.24 |
| Coagulation score, median [IQR] | 3 (1-3) | 2 (1-2) | 1.00 |
| Hepatic score, median [IQR] | 2 (0-2) | 0 (0-2) | 0.17 |
| Cardiovascular score, median [IQR] | 4 (3-4) | 4 (3-4) | 0.94 |
| Neurological score, median [IQR] | 0 (0-2) | 1 (0-1) | 0.85 |
| Renal score, median [IQR] | 1 (1-4) | 1 (0-3) | 0.51 |
| Total score, median [IQR] | 13 (8-16) | 9 (8-13) | 0.44 |
| Day 3, number (%) | 14 (78%) | 17 (94%) |  |
| Respiratory score, median [IQR] | 2 (2-2) | 2 (1-2) | 0.34 |
| Coagulation score, median [IQR] | 3 (1-3) | 2 (1-2) | 0.87 |
| Hepatic score, median [IQR] | 2 (0-2) | 0 (0-2) | 0.30 |
| Cardiovascular score, median [IQR] | 4 (3-4) | 4 (3-4) | 0.90 |
| Neurological score, median [IQR] | 0 (0-2) | 1 (0-1) | 0.72 |
| Renal score, median [IQR] | 1 (0-1) | 1 (0-3) | 0.97 |
| Total score, median [IQR] | 11 (6-12) | 10 (8-11) | 0.80 |
| Day 7, number (%) | 12 (67%) | 17 (94%) |  |
| Respiratory score, median [IQR] | 1 (1-2) | 1 (1-2) | 0.37 |
| Coagulation score, median [IQR] | 2 (0-2) | 1 (0-2) | 0.93 |
| Hepatic score, median [IQR] | 0 (0-2) | 0 (0-2) | 0.83 |
| Cardiovascular score, median [IQR] | 2 (0-3) | 1 (0-4) | 0.82 |
| Neurological score, median [IQR] | 0 (0-1) | 1 (0-1) | 0.23 |
| Renal score, median [IQR] | 0 (0-1) | 0 (0-3) | 0.35 |
| Total score, median [IQR] | 6 (3-9) | 6 (4-12) | 0.50 |

SOFA: sequential organ failure assessment, PMX-DHP-2h: polymyxin direct hemoperfusion for two hours, PMX-DHP-12h: polymyxin direct hemoperfusion for twelve hours, IQR: interquartile range
